# Responding to Unsolicited Medical Requests from Health Care Professionals on Pharmaceutical Industry-Owned Social Media Sites: Three Pilot Studies

**DOI:** 10.2196/jmir.9643

**Published:** 2018-10-29

**Authors:** Andrea M TenBarge, Jennifer L Riggins

**Affiliations:** 1 Medical Digital Strategy and Capabilities Eli Lilly and Company Indianapolis, IN United States

**Keywords:** social media, health care professionals, medical information

## Abstract

**Background:**

The use of social media has risen tremendously over the past decade with usage rates spanning from 5% American adults in 2005 up to 69% in 2016. A 2011 survey of 4033 clinicians found that 65% physicians use social media for professional purposes. To meet the changing needs and preferences of their customers, medical information departments within the pharmaceutical industry must continue to assess new digital channels such as social media and evolve their medical information services.

**Objective:**

The objective of the study was to pilot the use of social media as an additional channel to respond to unsolicited medical requests from health care professionals (HCP RUR) directed toward the pharmaceutical industry.

**Methods:**

From November 2016 to June 2017, 3 pilots were conducted during 3 professional congresses: the 2016 American College of Rheumatology Annual Meeting, the 2017 American Society of Clinical Oncology Annual Meeting, and the 2017 American Headache Society Annual Scientific Meeting. For each social media account, an identified community manager monitored the incoming account feed for proper triaging of posts. When an unsolicited medical request appeared, the community manager routed the question to the Tier One medical information contact center agents to respond. The following metrics were collected: total number of unsolicited requests directed to medical information contact center agents, total number of unsolicited requests that required escalation to Tier Two medical information associates, total number of unsolicited requests that were confirmed US HCPs, total number of unsolicited requests received after hours, and total number of unsolicited requests that were redirected to a different channel.

**Results:**

During the 3 pilots, 9 unsolicited medical requests were received with request numbers ranging from 2 to 4 requests per pilot. Of these, 1 was from a confirmed US HCP that required escalation to the Tier Two medical information associates. A majority of requests (7 out of 9) came in after the scheduled monitoring hours. There were 4 requests redirected to the medical information contact center phone number. The marketing accounts received more unsolicited medical requests than the corporate accounts (7 vs 2, respectively), and the 3 Twitter accounts saw more overall engagement (ie, medical requests and other general engagement) than the LinkedIn account.

**Conclusions:**

A limited number of medical questions were asked by confirmed HCPs using social media during the 3 pilots. New innovative medical information contact center channels often take time to build awareness. Continued channel awareness is needed to fully understand the channel’s desired use. Because consumers currently make up a majority of social media engagement, companies should look into creating a combined consumer and HCP RUR strategy to provide a better experience for all customers.

## Introduction

### The Evolution of Medical Information

Medical affairs organizations within the pharmaceutical industry act as the connecting link between research & development and commercialization teams. A core responsibility of Medical Affairs is to provide up-to-date, accurate, balanced, and nonpromotional responses to unsolicited medical requests regarding the company’s products. The action of responding to unsolicited requests (RUR) is performed by the Medical Affairs function, commonly referred to as medical information [1]. Over the past few decades, medical information departments have needed to evolve continually to meet the ever-changing digital landscape. Response channels used for RUR have ranged from basic channels such as the phone, fax, and hard copy in the 1980s and 1990s to the evolution of email responses and self-service websites in the 2000s [2]. Today, the world of digital channels has grown exponentially, leading to a focus on omnichannel response fulfillment with growing possibilities such as Web-based chat, texting, videoconferencing, podcasts, mobile apps, artificial intelligence, and social media. To meet the changing needs and preferences of their customers, medical information departments must continue to assess new digital channels for RUR, such as social media, and evolve their medical information services [2-3].

### Social Media Use and Health Care

The use of social media has risen tremendously over the past decade with usage rates spanning from only 5% of American adults in 2005 up to 69% in 2016. The most prevalent users continue to be the younger population with 86% of 18-29-year-olds using social media, followed by 80% of 30-49-year-olds, 64% of 50-64-year-olds, and 34% of those aged 65+ years [4]. A 2012 survey by PricewaterhouseCoopers’ Health Research Institute found that 1 in 3 consumers use social media for matters regarding their health with Facebook and YouTube being at the top. A majority of engagement comes from the younger population with 90% of 18-24-year-olds being likely to engage on social media or trust information for health matters compared with 56% of those aged 45-64 years [5].

An analysis of Twitter usage found over 100,000 health care professionals (HCPs) on Twitter in 2014, averaging 295,872 tweets per day. A majority of these HCPs were in the United States (45%), followed by Europe (22%), Near and Far East (17%), South America (13%), and Oceania (2%) [6]. A 2011 survey of 4033 clinicians found that 65% physicians use social media for professional purposes [7]. Furthermore, a 2014 survey of 350 nurse practitioners and physician assistants found that 45% of these HCPs also use social media for business purposes [8]. A search on LinkedIn in November 2017 detected over 400,000 profiles using the term “pharmacist,” over 450,000 profiles using the term “physician,” and over 1,647,000 profiles using the term “nurse” [9]. Instances of professional use include networking, crowdsourcing, sharing and consuming information, curating information, educating the public, patient engagement and feedback, and discussing health care policy [10-14].

Furthermore, Twitter has increasingly been used during medical conferences to share and discuss information related to the conference [10,13]. Ample literature has been published looking at the role of Twitter at various conferences across specialties [15-22]. A tweet analysis of 13 conferences from 2011-2013 identified 51,159 tweets by 8778 Twitter account holders of which 25% were identified HCPs who composed 19,503 (38%) of the tweets [15].

### Social Media and the Pharmaceutical Industry

In recent years, pharmaceutical companies have joined the social media revolution, creating their own corporate or marketing accounts [23-25]. A study of 15 of the top pharmaceutical companies from October 1, 2013 to September 30, 2014 found that 93% of the companies had a company-owned Twitter page, followed by 66% for YouTube, 66% for Facebook, and 60% for LinkedIn. These pages averaged approximately 45,000 subscribers or followers, and a majority of the posts were related to company news (63.4%) and help-seeking electronic direct-to-consumer advertising (40.7%) [23]. In 2014, the Food and Drug Administration (FDA) put out 3 draft guidance documents for industry to assist companies in developing their strategy for participation in social media [26-28].

In the 2011 FDA draft guidance on RUR for off-label information, the FDA states that because other responders in public forums often do not have the most up-to-date and robust information that pharmaceutical companies do on their products, it may be in the public’s best interest for the company to respond to unsolicited requests regarding their products in these channels [29]. Most pharmaceutical companies, however, have yet to take full advantage of the customer service capabilities that the medical side of the organization can offer on social media [25]. Those that have ventured into this area often do so by redirecting the customer to the company’s contact center number when an unsolicited request for information is received [30]. There are 2 companies that have attempted to incorporate medical information services into their social media strategy by partnering with Sermo to allow physicians to submit unsolicited medical requests to the company [31]. Sermo is a global physician-only social media platform that includes over 800,000 physicians from 150 countries around the world [32]. Survey results showed that 73% of the Sermo physicians found direct access to the company’s medical expert as valuable or extremely valuable [31].

### Social Media Business Case Development

Currently, medical information services are provided through a number of traditional channels such as telephone, email, live chat, and a self-service website. When evaluating the addition of innovative digital channels to the omnichannel nature of medical information services, there is often a lack of information in the primary literature to assist pharmaceutical companies in developing their strategy. Therefore, to fully assess the channel’s potential desired use, proof of concept tactics such as market research are frequently utilized before moving forward with a pilot.

From September 2, 2016 to October 3, 2016, a third-party vendor was contracted to initiate, draft, and complete a market research survey analyzing HCP use of social media for medical information. The Web-based survey was completed by 100 HCPs consisting of 50 physicians and 50 allied HCPs. Survey results showed that a majority of physicians (41/50, 82%) and allied HCPs (45/50, 90%) had asked a medical or product-related question on social media before. Furthermore, a majority of physicians (32/50, 64%) and allied HCPs (36/50, 72%) feel that social media is somewhat, very, or extremely valuable for interacting with pharma. Therefore, with the market research providing a positive business case for piloting HCP RUR on social media, the decision was made to move forward to the pilot stage. The objective of the following research is to pilot the use of social media as an additional channel to respond to unsolicited medical requests from HCPs (HCP RUR) directed toward the pharmaceutical industry.

## Methods

### Pilot Overviews

From November 2016 to June 2017, 3 pilots were conducted covering 3 professional congresses: the 2016 American College of Rheumatology Annual Meeting, the 2017 American Society of Clinical Oncology Annual Meeting, and the 2017 American Headache Society Annual Scientific Meeting (Table 1). Identified social media accounts were limited to those that were company-owned and had HCPs as one of their target audiences. Corporate communications- and therapeutic area-focused accounts that were active during the congresses were prioritized. The social media account owners were required to have a statement on the page that let the audience know how the company would engage on the site. The account owners were also required to have a process in place for handling and reporting adverse events and product complaints, a process for handling misinformation, a process for crisis management, and a triage process in place to ensure requests were directed to the appropriate groups for responding. Engagement tool selection was based on the current engagement tool that the account owner was using. The 2 engagement tools utilized were Spredfast, a social marketing platform (headquartered in Austin, TX), and Social Studio, a product of the Salesforce Marketing Cloud (headquartered in San Francisco, CA).

### Process for Responding to Unsolicited Medical Requests from Health Care Professionals

**Figure 1:**
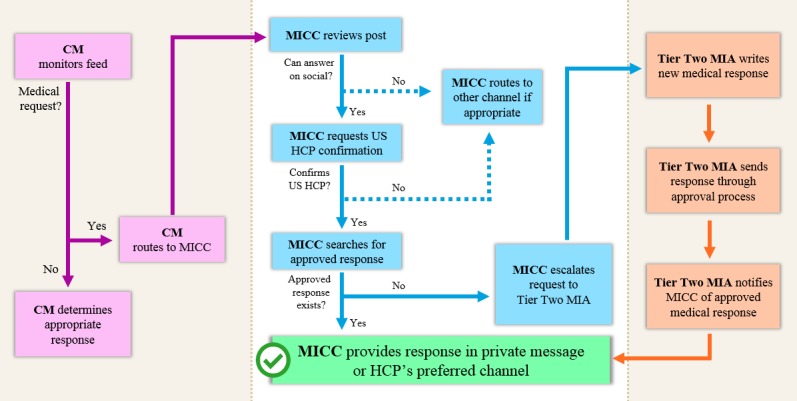
Process for responding to unsolicited medical requests from health care professionals. CM: community manager; MICC: medical information contact center; HCP: health care professional; MIA: medical information associate.

For each social media account, an identified community manager monitored the incoming account feed for proper triaging of posts (Figure 1). When an unsolicited medical request was directed to the company-owned social media account, the community manager would route the question to the Tier One medical information contact center agents to respond. If the question was deemed answerable with a medical response, the medical information contact center agents would direct the requestor to a private message and ask for confirmation that the requestor was a US HCP. If the requestors confirmed they were US HCPs, the medical information contact center agents would then provide the answers within a private message conversation. If the appropriate medical answer was available on the company’s medical information website, medical information contact center agents would provide links to the medical answer within the private message response. This ensured the answers would always remain current and would contain appropriate regulatory information. If an appropriate medical answer was not currently available, the medical information contact center agents would escalate the question to the Tier Two medical information associates for creation and approval of a medical response to answer the question. Due to licensing fees and the limited timeframes of Pilots 1 and 2, a Medical Digital Strategy Consultant acted as a liaison between the medical information contact center agents and the Spredfast engagement tool to ensure proper posting of responses.

In preparation for the pilots, training and simulation sessions were held to teach key participants the process and guardrails for HCP RUR on social media. Subject matter experts were on call to assist with strategy-related questions that occurred during the pilot, including subject matter experts for medical social media operations, contact center operations, social media account operations, and engagement tool operations.

**Table 1:** Overview of pilots.

Features	Pilot 1	Pilot 2	Pilot 3
Congress	2016 American College of Rheumatology Annual Meeting	2017 American Society of Clinical Oncology Annual Meeting	2017 American Headache Society Annual Scientific Meeting
Pilot dates	November 11-16, 2016	June 2-6, 2017	June 1, 2017-ongoing
Pilot hours	9 am-5 pm EST	9 am-8 pm EST	Ongoing: 9 am-5 pm EST weekdays; Congress: 9 am-8 pm EST
Social accounts	US Rheumatology Twitter handle	Main corporate Twitter handle and US Oncology LinkedIn account	US Migraine Twitter handle
Account owner	Marketing	Corporate	Marketing
Engagement tool	Spredfast	Spredfast	Social Studio

### Pilot Metrics

To evaluate the pilots and inform shared learning for future process improvements, the following metrics were identified and collected:

- total number of unsolicited requests directed to medical information contact center agents
- total number of unsolicited requests that required escalation to Tier Two medical information associates
- total number of unsolicited requests that were confirmed US HCPs
- total number of unsolicited requests received after hours
- total number of unsolicited requests that were redirected to a different channel

## Results

### Pilot 1

**Figure 2:**
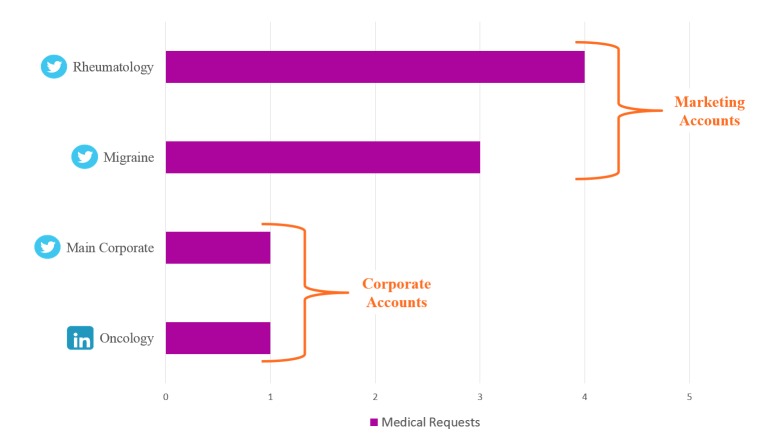
Total medical requests by social media account.

During Pilot 1, 4 unsolicited medical requests were received (Table 2 and Figure 2). Of these, 1 was a US HCP asking about registration for a congress event that was to occur the following day. This request required escalation to the Tier Two medical information associate to draft and approve a custom response. Due to the extra effort needed, a response was not able to be provided until shortly before the event occurred. Another was a consumer asking about pipeline information for a specific indication. This requestor was not a US HCP, so an approved pleasantry was used as the response. Even though the question could not be answered, the consumer provided positive feedback, thanking the team for listening. The third question asked for clarification regarding a scientific term in one of the posts. This question was answered correctly by an external Twitter user before the team could respond. The fourth question asked about disease control. However, the requestor never responded to the tweet asking for US HCP confirmation. Of the 4 unsolicited requests, 3 were received after designated monitoring hours.

### Pilot 2

During Pilot 2, 2 unsolicited medical requests were received (Table 2 and Figure 2). Of these, 1 request was received on the US Oncology LinkedIn account and inquired about drug targeting. Due to the inability to private message between individuals and company pages on LinkedIn, the requestor was directed to the medical information contact center phone number. The second request was received after designated monitoring hours on the company’s main corporate Twitter handle. The requestor asked about medication cost and was directed to the medical information contact center phone number owing to the complexity of the response.

**Table 2:** Medical request metrics by pilot.

Metrics	Pilot 1, n	Pilot 2, n	Pilot 3, n	Total, n
Total medical requests	4	2	3	9
Tier two requests	1	0	0	1
Confirmed US health care professional requests	1	0	0	1
After hours requests	3	1	3	7
Requests redirected to call number	0	2	2	4

### Pilot 3

During the first 3 weeks of Pilot 3, 3 unsolicited medical requests were received (Table 2 and Figure 2). Of these, 2 were directed to the medical information contact center phone number (1 asking about nondrug treatment and the other asking about general disease state help). The third question asked about diagnostic imaging. A tweet was sent asking for US HCP confirmation, but the requestor did not reply. All 3 requests came in after designated monitoring hours. No unsolicited medical requests were received during the American Headache Society Annual Scientific Meeting.

## Discussion

### Pilot Comparison of Request Volume

During the 3 pilots, 9 unsolicited medical requests were received, with medical request numbers ranging from 2 to 4 requests per pilot (Table 2). Pilot 1, during the 2016 American College of Rheumatology Annual Meeting, saw the most activity with 4 total medical requests and 1 Tier Two request. Pilot 2, during the 2017 American Society of Clinical Oncology Annual Meeting, saw the least activity with only 2 total medical requests. All 3 of the requests received during Pilot 3 came in during the first month.

### Account Comparison of Request Volume

Of the 4 social media accounts used during the 3 pilots, 2 were owned by Marketing, and 2 were corporate-owned accounts (Figure 2). The marketing accounts received more unsolicited medical requests than the corporate accounts (7 vs 2, respectively). This could be owing to the marketing accounts posting more scientifically detailed material while the corporate accounts posted higher level, corporate-focused information. The 3 Twitter accounts saw more overall engagement (ie, medical requests and other general engagement) than the LinkedIn account, which only received 1 medical request. This may be owing to most of the pilots occurring during a medical congress, where Twitter activity tends to increase with the use of congress-specific hashtags.

### Hours of Operation

Normal medical information contact center hours of operation are from 9 am-8 pm EST Monday through Friday. However, the 9 am-5 pm EST monitoring timeframe was chosen for Pilot 1 owing to the scheduled working hours for the community managers. During Pilot 1, 3 of the 4 unsolicited medical requests that were received were after the scheduled monitoring hours of 9 am-5 pm EST. As a result, hours of operation were expanded during Pilots 2 and 3 to cover 9 am-8 pm EST during congress days, when activity was expected to be higher while maintaining the 9 am-5 pm EST working hours during standard business weekdays. Even with the expanded hours, a majority of the requests continued to come in after scheduled monitoring hours during Pilots 2 and 3.

### Health Care Professional Versus Consumer Requests

Only 1 of the 9 unsolicited medical requests was from a confirmed US HCP. The other requests were from potential consumers or did not respond to the tweet asking them to confirm they were a US HCP. Although the capability was intentionally set up for US HCPs, much of the engagement on the sites appeared to be from consumers. Having a parallel capability setup for answering medical requests from US consumers in addition to US HCPs would provide a better experience for all customers.

### Social Media Question Nuances and Preparation

With the addition of new digital channels comes the potential for new types of questions owing to the nature of the channels. Since HCP use of social media, especially Twitter, tends to increase around medical congresses, congress logistics questions may be more prominent. These questions can be more time-sensitive, as experienced in the pilot, as opposed to questions received in other HCP RUR channels. Having approved responses prepared for standard congress logistics questions would help minimize the response time to the customer while also minimizing the workload of the medical information personnel. Another item to consider when preparing for potential questions is the type of information that is planned to be presented or released during the congress, as this information is more likely to appear in social media discussions.

### Awareness of the Capability

When adding new innovative medical information contact center digital channels, awareness is key. Awareness often takes months to years to build up enough responsiveness to see the true desired use of a channel. As was found in the market research proof of concept, a majority of HCPs, who said they have never asked a medical or product-related question on social media, said it was because they simply had not thought to ask this type of question on social media. However, 71% (10/14) of these nonusers said they would consider using social media to ask a medical information question to a pharmaceutical company in the future. Although most retail companies in the consumer market have a large customer service representation on social media, the pharmaceutical industry has been slow to adopt this capability owing to the highly regulated nature of their environment. Many pharmaceutical companies simply refer requestors to their contact center phone number, if they respond at all. Therefore, HCPs may not think their question will be answered if they submit a request to pharma on social media. However, to aid in awareness of the social media pilots, engagement guidelines (including items such as hours of operation) were published on participating social media account pages through a pinned tweet or within the guidelines section of the page. The new social media channel capability was also included within a larger medical-wide awareness campaign, which provided awareness regarding the medical organization and the various channels HCPs may use to ask a medical question.

### Limitations

During Pilots 1 and 2, medical information contact center agents did not have direct access to the social media engagement tools owing to the limited timeframe of the pilots. This, in turn, added extra steps to the RUR workflow, which increased the overall response time to the requestor. In addition, pilot metrics included information to aid in pilot strategy and uptake but were limited in helping to gauge the overall response quality. Satisfaction surveys can be critical to help strategy leaders fully understand the quality of the capability. Although the pilots offer great insights into the incorporation of HCP RUR on Twitter, every social media platform tends to have its specific uses and features; for example, LinkedIn does not allow private messaging between company pages and individuals. Additional research needs to be conducted to understand the differences in how a company can respond using various platforms such as Facebook, Sermo, Doximity, etc. Furthermore, these 3 pilots were US-focused. Because regulations, digital channel usage, and digital platforms often vary by country, further research is needed to better understand the nuances in setting up social media RUR capabilities in countries around the world.

### Conclusions

The 3 HCP RUR social media pilots produced 9 unsolicited medical requests. While this number is considered low for more traditional channels such as the phone, the new innovative medical information contact center channels often take time to build up an increased level of awareness of the new service offering. Providing channel awareness is critical to fully understand the channel’s true desired use. In addition, because consumers currently appear to make up a majority of social media engagement, companies should look into creating a combined consumer and HCP RUR strategy that can be carried out consistently across sites. In conclusion, the pilots provided pertinent strategy insights such as the initial volume to expect and resources required when developing a social media strategy for HCP RUR.

## Abbreviations

FDA: Food and Drug Administration
HCP: health care professional
RUR: responding to unsolicited requests
